# Supplementary figures and images for: Changes in lipid metabolism track with the progression of neurofibrillary pathology in tauopathies
Source: J Neuroinflammation. 2024 Mar 27;21:78. doi: 10.1186/s12974-024-03060-4 (PMC10976809; doi:10.1186/s12974-024-03060-4)

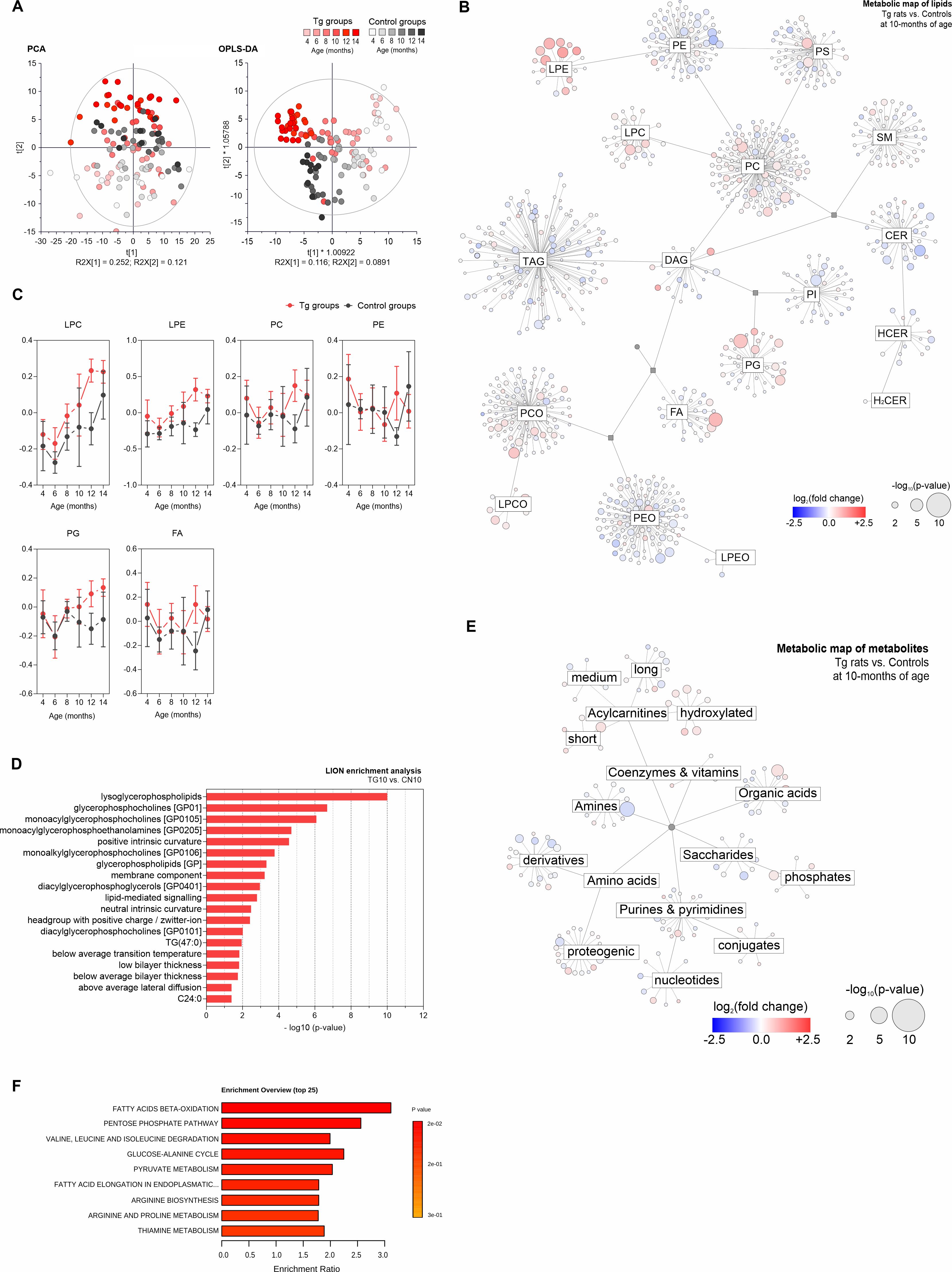

Supplement: Supplementary file 6 — Supplementary Material 6 [file 12974_2024_3060_MOESM6_ESM.jpg]

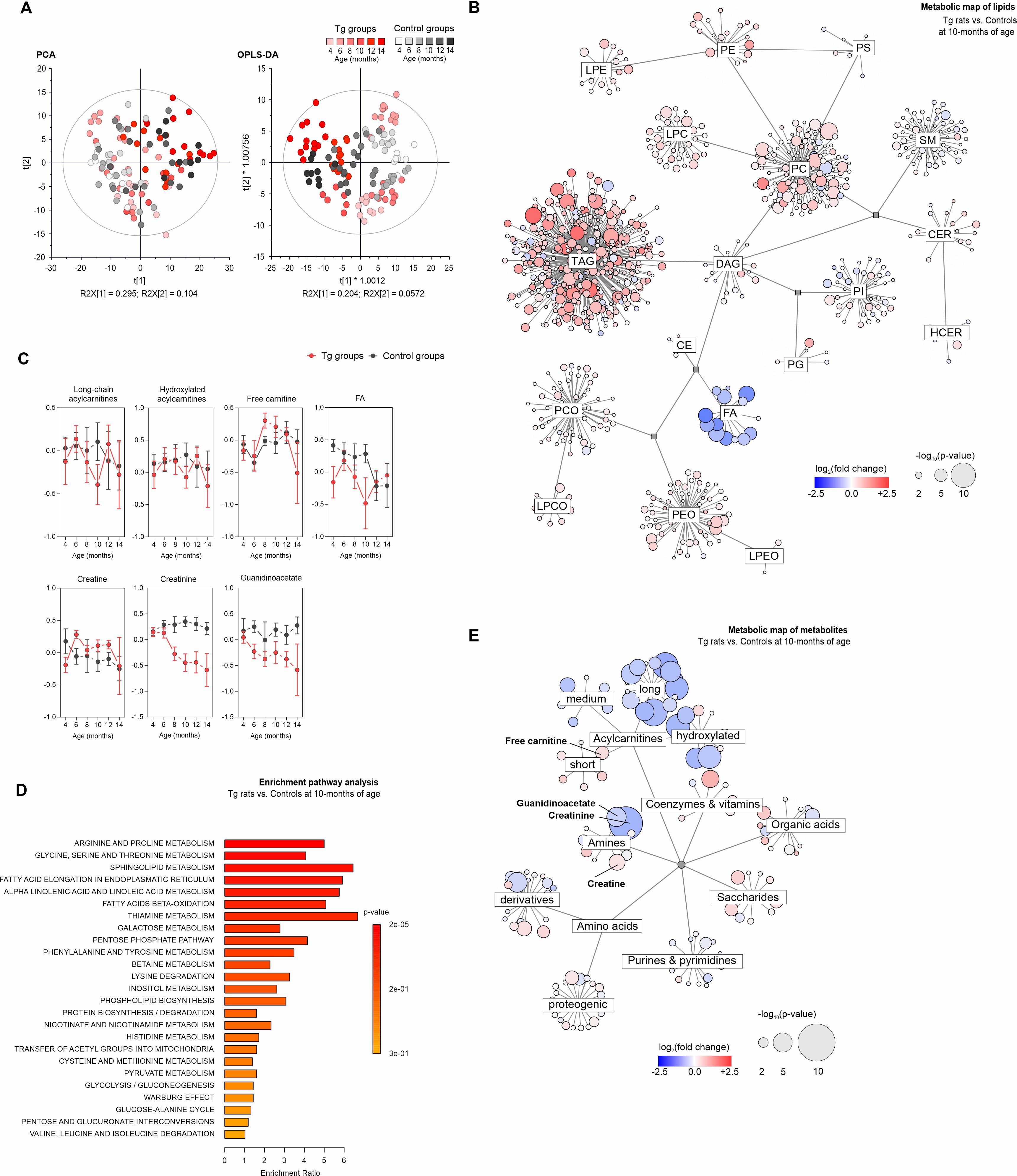

Supplement: Supplementary file 7 — Supplementary Material 7 [file 12974_2024_3060_MOESM7_ESM.jpg]

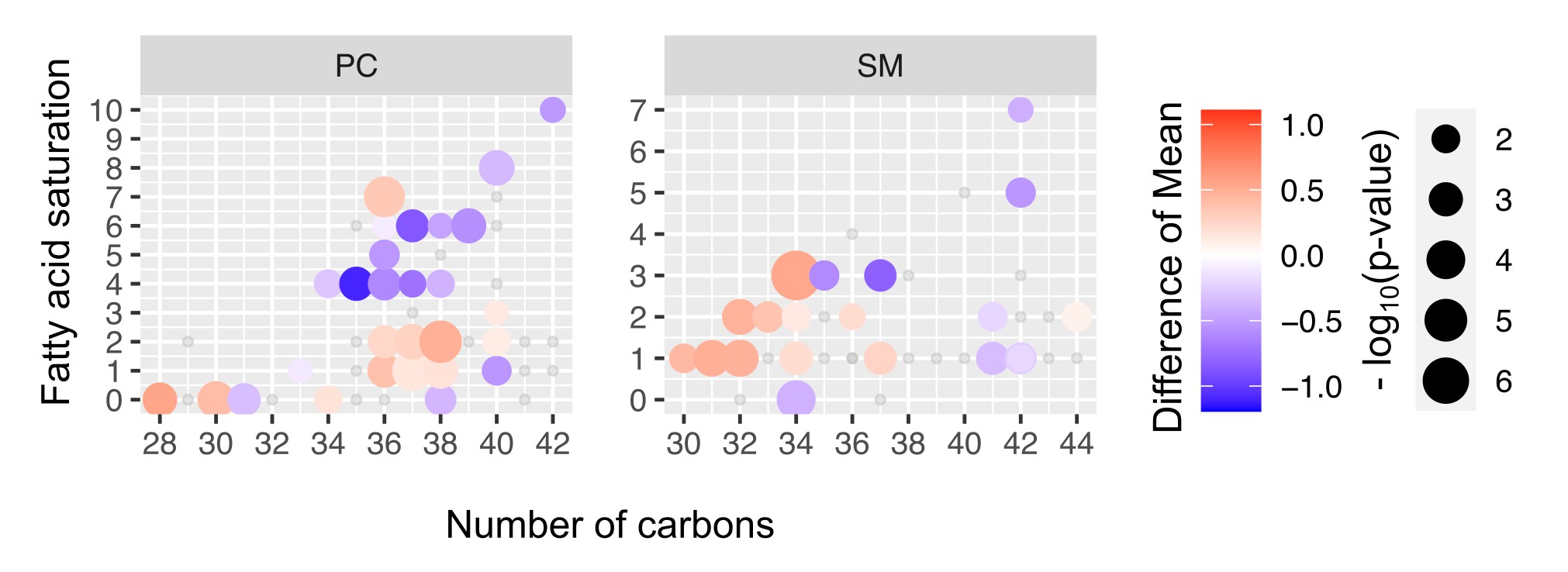

Supplement: Supplementary file 8 — Supplementary Material 8 [file 12974_2024_3060_MOESM8_ESM.jpg]

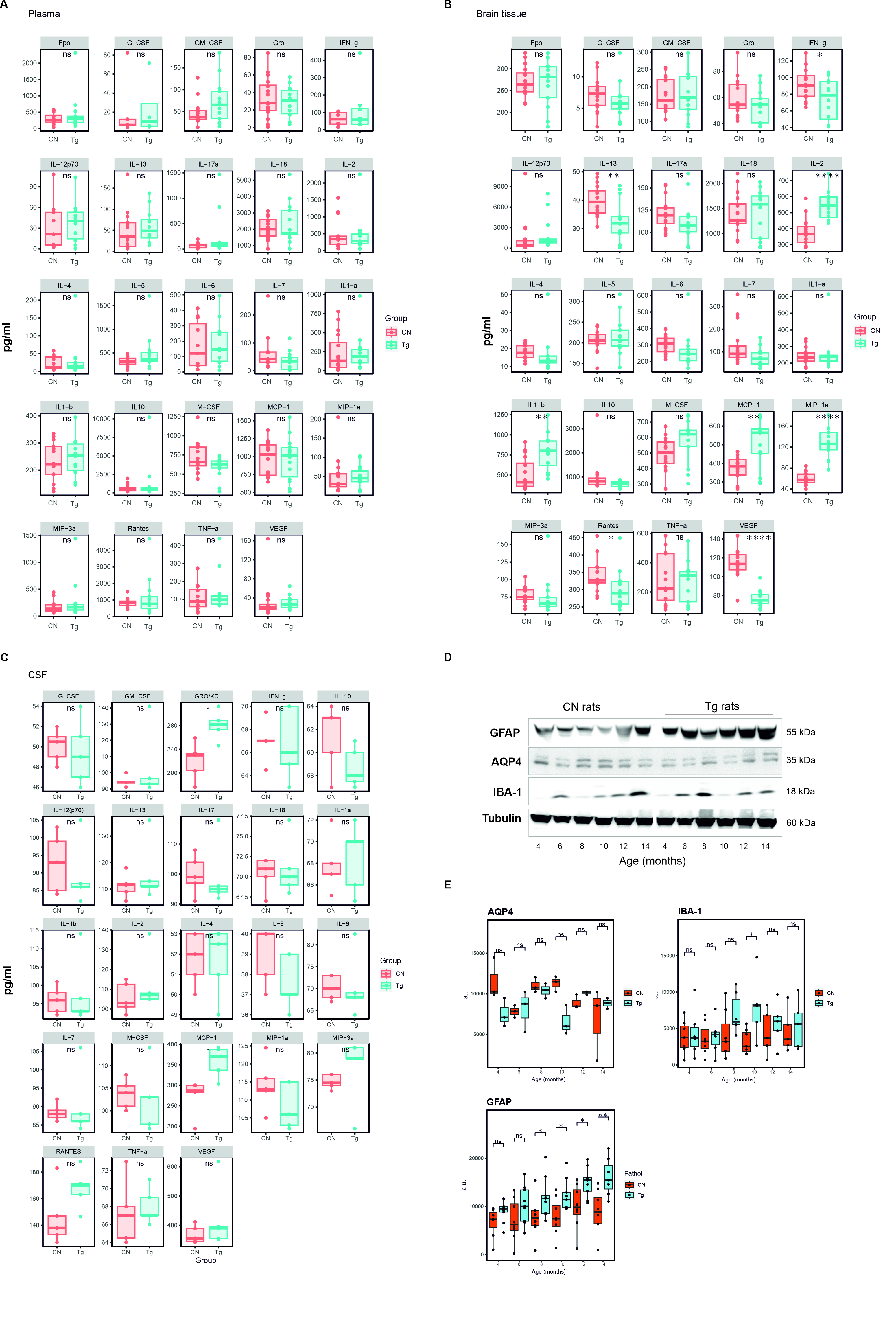

Supplement: Supplementary file 9 — Supplementary Material 9 [file 12974_2024_3060_MOESM9_ESM.jpg]
