# Supplementary material for: Changes in lipid metabolism track with the progression of neurofibrillary pathology in tauopathies
Source: J Neuroinflammation. 2024 Mar 27;21:78. doi: 10.1186/s12974-024-03060-4 (PMC10976809; doi:10.1186/s12974-024-03060-4)
Supplement: Supplementary file 10 — Supplementary Material 10 [file 12974_2024_3060_MOESM10_ESM.docx]

**Supplementary information**

**Changes in lipid metabolism track with the progression of neurofibrillary pathology in tauopathies.**

Dominika Olešová^1†^, Dana Dobešová^2†^, Petra Majerová^1^, Radana Brumarová^2^, Aleš Kvasnička^2^, Štěpán Kouřil^2^, Eva Stevens^1^, Jozef Hanes^1^, Ľubica Fialová^1^, Alena Michalicová^1^, Juraj Piešťanský^1^, Jakub Šinský^1^, Petr Kaňovský^3,4^, David Friedecký^2^*, Andrej Kováč^1^*

^1^ Institute of Neuroimmunology, Slovak Academy of Sciences, Dúbravská cesta 9, 845 10, Bratislava, Slovak Republic

^2^ Laboratory for Inherited Metabolic Disorders, Department of Clinical Biochemistry, University Hospital Olomouc and Faculty of Medicine and Dentistry, Palacký University Olomouc, Zdravotníků 7, 779 00 Olomouc, Czech Republic

^3^ Department of Neurology, University Hospital Olomouc, Zdravotníků 7, 779 00, Olomouc, Czech Republic

^4^ Department of Neurology, Faculty of Medicine and Dentistry, Palacky University, Zdravotníků 7, 779 00, Olomouc, Czech Republic

**Figure S1 - Metabolic patterns of brain tissue affected by neurofibrillary pathology.** Univariate and multivariate analyses of metabolic patterns were found in the pons of 10-month-old SHR-24 Tg rats compared with age-matched controls (SHR). **A** Principal component analysis (PCA) and orthogonal partial least square discriminant analysis (OPLS-DA) **B** Metabolic map showing results from significance testing for lipids (student's t-test and fold change). The size of each bubble represents the statistical significance (p-value), and color and shade indicate the level of fold change between SHR-24 Tg rats and control groups. **C** Age-related changes of the most distinctive lipid classes (data are presented as median intensities with 95% confidence intervals). **D** Lipid ontology (LION) enrichment analysis. **E** Metabolic map showing results from significance testing for metabolites (student's t-test and fold change). The size of each bubble represents the statistical significance (p-value), and color and shade indicate the level of fold change between SHR-24 Tg rats and control groups. **F** Enrichment pathway analysis of metabolomics data. The biochemical pathways were assessed using statistical significance (p-value) and enrichment ratio.

Explanation of lipid class abbreviations: LPE – lysophosphatidylethanolamines, PE – phosphatidylethanolamines, PS – phosphatidylethanolserines, PC – phosphatidylcholines, LPC – lysophosphatidylcholines, SM – sphingomyelins, CER – ceramides, HCER – hexosyl-ceramides, H2CER – dihexosyl-ceramides, DAG – diacylglycerols, TAG – triacylglycerols, PI – phosphatidylinositols, PG – phosphatidylglycerols, CE – cholesteryl esters, FA – fatty acids, PCO – plasmenyl phosphatidylcholines, LPCO – plasmenyl lysophosphatidylcholines, PEO – plasmenyl phosphatidylethanolamines, LPEO – plasmenyl lysophosphatidylethanolamines.

**Fig. S2 - Global effects of brain aberrant metabolism on the composition of plasma.** Univariate and multivariate analyses of metabolic patterns found in the plasma of 10-month-old SHR-24 Tg rats compared with age-matched controls. **A** Principal component analysis (PCA), and orthogonal partial least square discriminant analysis (OPLS-DA). **B** Metabolic map showing results from significance testing for lipids – student's t-test (p-value and fold change). **C** Age-related changes of the most distinctive lipid classes (data are presented as median intensities with 95% confidence intervals). **D** Enrichment pathway analysis of metabolomics data. The biochemical pathways were assessed using statistical significance (p-value) and enrichment ratio. **E** Metabolic map showing results from significance testing for metabolites (student's t-test and fold change).

Explanation of lipid class abbreviations: LPE – lysophosphatidylethanolamines, PE – phosphatidylethanolamines, PS – phosphatidylethanolserines, PC – phosphatidylcholines, LPC – lysophosphatidylcholines, SM – sphingomyelins, CER – ceramides, HCER – hexosyl-ceramides, H2CER – dihexosyl-ceramides, DAG – diacylglycerols, TAG – triacylglycerols, PI – phosphatidylinositols, PG – phosphatidylglycerols, CE – cholesteryl esters, FA – fatty acids, PCO – plasmenyl phosphatidylcholines, LPCO – plasmenyl lysophosphatidylcholines, PEO – plasmenyl phosphatidylethanolamines, LPEO – plasmenyl lysophosphatidylethanolamines.

**Fig. S3 – Composition of PC and SM lipid classes.** Fatty acid composition of selected lipid classes. Student's t-test corrected on multiple comparisons by Bonferroni and difference of mean.

Explanation of lipid class abbreviations: PC – phosphatidylcholines, SM – sphingomyelins.

**Fig. S4 - Inflammatory states of glial cells in relation to tau pathology in transgenic SHR-24 rat model for tau pathology.** Quantitative Analysis of Cytokine and Chemokine Levels in Plasma, CSF, and Brain Tissue (A-C) present the measured concentrations of cytokines and chemokines in the plasma (A), brainstem (B), and cerebrospinal fluid (CSF). The levels of glial fibrillary acidic protein (GFAP), aquaporin 4 (AQP4), and ionized calcium-binding adaptor molecule 1 (Iba-1) in brain tissue were monitored by Western blot analysis (D and E)

**Table S1 –** LION-term enrichment analysis of 10 – 14-month-old Tg vs Cn animals. Enrichment analyses of lipidomic data of medulla, pons, and CSF of 10, 12, and 14-month-old groups in the "target-list mode".

**Table S2 –** Quantitative analysis of Neurofilament light chain in plasma and total Tau in the CSF.

**Table S3 –** Heatmaps showing results from significance testing of Tg vs. Control groups and the aging process in both Tg and Control groups.

**Table S4 –** Summary of lipids and metabolites identified in medulla, pons, CSF, and plasma of Tg and Control rats, including nomenclatures used for metabolomics.

**Table S5 –** Metabolic set enrichment analysis showing the altered pathways and metabolites revealed in medulla, pons, CSF, and plasma
